# A novel TP53 variant (rs78378222 A > C) in the polyadenylation signal is associated with increased cancer susceptibility: evidence from a meta-analysis

**DOI:** 10.18632/oncotarget.9056

**Published:** 2016-04-27

**Authors:** Ying Wang, Xue-Song Wu, Jing He, Tianjiao Ma, Wei Lei, Zhen-Ya Shen

**Affiliations:** ^1^ Department of Cardiovascular Surgery, The First Affiliated Hospital and Institute for Cardiovascular Science, Soochow University, Suzhou, Jiangsu, China; ^2^ School of Humanities and Social Science, Harbin Medical University, Harbin, Heilongjiang, China; ^3^ Department of Pediatric Surgery, Guangzhou Institute of Pediatrics, Guangzhou Women and Children's Medical Center, Guangzhou Medical University, Guangzhou, Guangdong, China; ^4^ Department of Experimental Research, Collaborative Innovation Center for Cancer Medicine, State Key Laboratory of Oncology in South China, Sun Yat-Sen University Cancer Center, Guangzhou, Guangdong, China; ^5^ Department of Internal Medicine, Harbin Medical University, Harbin, Heilongjiang, China

**Keywords:** TP53, rare variant, GWAS, apoptosis, polymorphism

## Abstract

Polymorphisms in TP53 are involved in the progression of different types of cancer. A rare novel TP53 variant (rs78378222 A > C allele) was found via whole-genome sequencing in 2011. This variant was shown to significantly increase the risk of glioma, colorectal adenoma and prostate cancer. Functional analysis further revealed that this variant hindered TP53 expression and its downstream effect on apoptosis. Several studies have investigated the relationship between rs78378222 and cancer susceptibility. However, the results were not consistent. We conducted the first meta-analysis to give a more credible assessment on the association about this variant and cancer risk. Our meta-analysis included 34 studies consisting of 36599 cases and 91272 controls. These studies were mostly on the basis of high-grade data from Genome-wide association studies (GWASs). The results indicated that TP53 rs78378222 was significantly associated with an increased risk of overall cancer (AC vs. AA: OR = 1.511, 95% CI = 1.285–1.777). Furthermore, stratified analyses indicated that rs78378222 increased the risk of nervous system cancer, skin cancer and other cancer. To summarize, this meta-analysis suggested that rs78378222 C allele is a potent risk factor for overall cancer.

## INTRODUCTION

Cancer has become an important challenge to public health. According to the GLOBOCAN 2012, approximately 14.1 million new cancer cases and 8.2 million cancer deaths were reported worldwide [1]. High frequency of TP53 mutations was found in many types of human cancer [2]. The protein product-p53, comprised of 394 amino acid residues, is a versatile protein involved in genome stability, DNA repair, apoptosis, cell cycle arrest and senescence [3]. Over-expressing of p53 alone was sufficient to shrink the tumor volume in mice [4, 5].

In the past decades, many researches focused on the p53 coding sequence (CDS) mutations, especially Li-Fraumeni mutations, which resulted in mutant p53 proteins that lacked normal functions and conferred oncogenic properties [6]. The function studies indicated that the p53 protein variant (72Pro/Pro) was likely to induce apoptosis with decreased kinetics, when compared with wild-type P53 (72Arg/Arg) [7]. But mouse models demonstrated that some genetic variations in TP53 enhancing oncogenic potential could not be simply attributed to defection of p53 CDS [8, 9].

Recently, numerous next-generation sequencing data of paired tumor-normal genomes revealed several striking rare mutations [10, 11]. It was found that rare variants had a more important functional effect than common variants [12, 13]. They might contribute to the inherited predisposition to cancer [14]. A GWAS reported a novel rare variant (rs78378222 A > C) in the polyadenylation signal sequence of *TP53*, which was associated with increased risk of several cancers [15]. The A-to-C transition leads to the change from AATAAA polyadenylation signal to AATACA. It causes the formation of a impaired *TP53* 3′ 2-end processing, thereby decreasing *TP53* expression levels (*P* = 0.041) [15]. Moreover, this variant also hinders the p53-induced apoptosis [3].

In the GWAS containing 16 million SNPs identified from whole-genome sequencing, authors found the strongest signal from rs78378222 (OR = 2.36, *P* = 5.2 × 10^−17^) [15]. This A-to-C polymorphism significantly increased the risk of prostate cancer, glioma and colorectal adenoma among Caucasians in European and the United States [15]. Since then, many investigations were conducted to assess the association between rs78378222 polymorphism and cancer susceptibility. But the results were inconclusive, especially by ethnicity and the types of cancer. Guan et al. conducted a mini meta-analysis on the association between rs78378222 and overall cancer risk, but included only 11 studies and analyzed simply [16]. To provide a precise evaluation of the association of interest, we performed this comprehensive meta-analysis via including all the eligible studies.

## RESULTS

### Study characteristics

**Figure 1:**
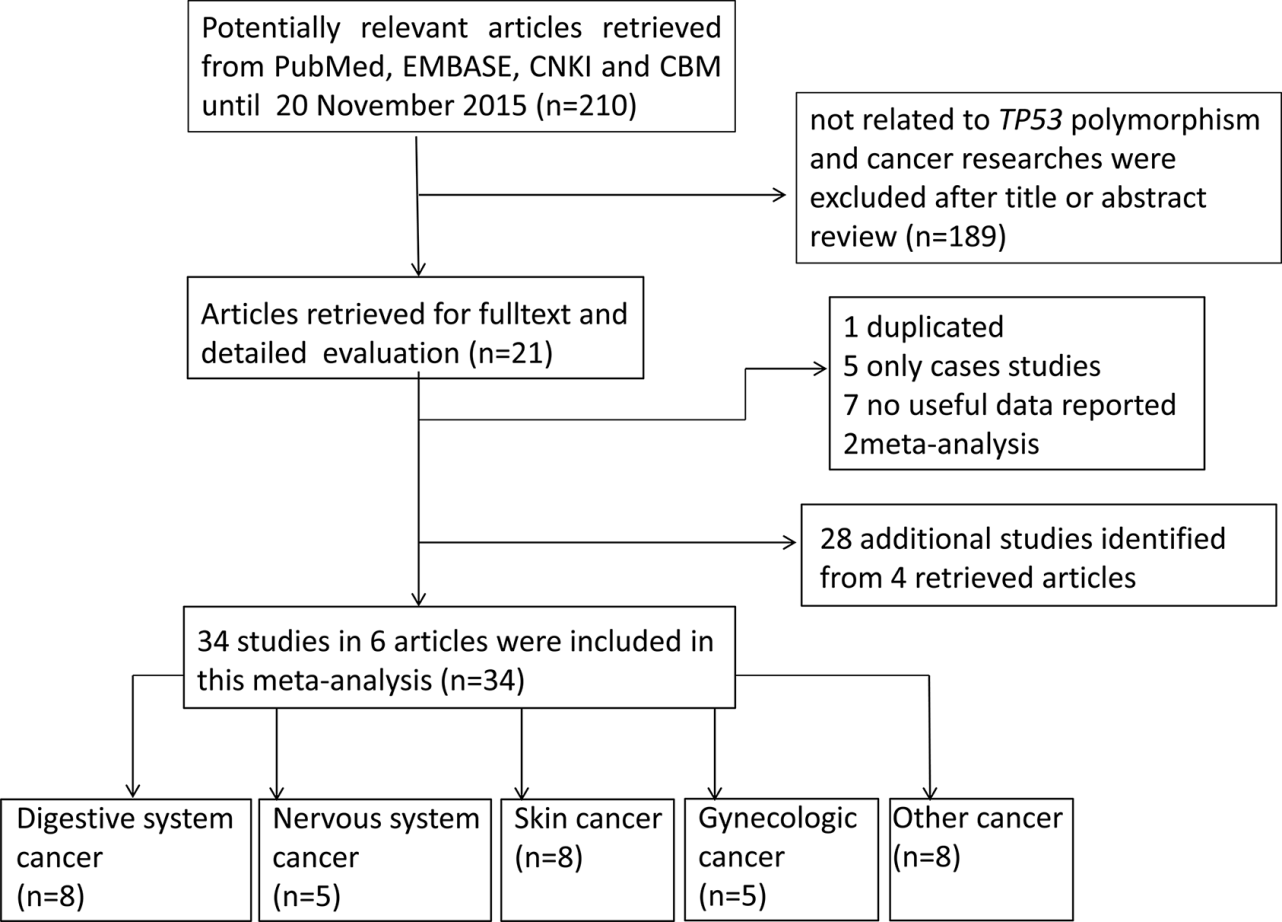
Flowchart of included studies for the meta-analysis of the association between *TP53* rs78378222 polymorphism and overall cancer risk

We retrieved the literatures from PubMed and EMBASE using the search terms described in methods section without language restriction. We first excluded 189 publications not concerning the TP53 polymorphism and cancer after a title and abstract screening. Then the remaining 21 articles were carefully full-text reviewed. As a result, 15 publications were further removed for the following reasons:1 study was duplicated with study included in the meta-analysis; 5 were case only studies;7 had no adequate data to calculate OR and 95% CI; 2 were meta-analysis. Finally, only 6 studies were included in the final analysis (Figure 1) [15–20]. Moreover, we retrieved 34 separated investigations from 4 studies [15–18]. After all the steps of literature review, 34 studies including 36599 cases and 91,272 controls were ultimately included in our meta-analysis (Table 1). Among them, there were 8 studies on digestive system cancer, 5 on nervous system cancer, 8 on skin cancer, 5 on gynecologic cancer, 8 on other cancer. Moreover, 6 studies were considered as low quality (quality score < 10), and 28 studies were considered as high quality (quality score ≥ 10). In the included studies, all the cancer cases were histologically confirmed, and controls were matched to cases by sex, age and ethnicity in 24 studies.

**Table 1:** Characteristics of the 34 studies included in this meta-analysis for the association between rs78378222 and cancer risk

Surname	Year	Country	Ethnicity	Cancertype	Control source	Genotypingmethods	Cases	Controls	MAF(T)	Score	HWE
Rao	2014	India	Indian	Oralcancer	PB	PCR-RFLP	96	504	0.000	6	/
Rao	2014	India	Indian	Cervicalcancer	PB	PCR-RFLP	108	504	0.000	6	/
Rao	2014	India	Indian	Breastcancer	PB	PCR-RFLP	235	504	0.000	6	/
Diskin	2014	USA	Caucasion	Neuroblastoma	HB	microarray	2,436	4,955	0.011	7	0.42
Diskin	2014	USA	African	Neuroblastoma	HB	microarray	365	2,491	0.002	7	0.92
Guan	2013	USA	Caucasian	melanoma	HB	Taqman assay	1,329	1,298	0.013	11	0.64
Guan	2013	USA	Caucasian	SCCHN	HB	Taqman assay	1,096	1,086	0.014	11	0.63
Guan	2013	USA	Caucasian	lungcancer	HB	Taqman assay	1,013	1,074	0.012	11	0.69
Egan	2012	USA	Caucasian	glioma	PB	Taqman assay	566	603	0.011	10	0.79
Zhou	2012	China	Asian	esophageal carcinoma	PB	PCR-RFLP	405	810	0.010	8	0.78
Stacey	2011	Iceland	Caucasian	BCC	PB	PCR-RFLP	2,857	43,909	0.019	12	/
Stacey	2011	Denmark	Caucasian	BCC	PB	PCR-RFLP	308	3,441	0.017	12	0.31
Stacey	2011	Eastern Europe	Caucasian	BCC	PB	PCR-RFLP	526	532	0.007	12	0.88
Stacey	2011	Spain	Caucasian	BCC	PB	microarray	628	3,928	0.005	12	0.77
Stacey	2011	Iceland	Caucasian	Prostate cancer	PB	microarray	3,306	43,531	0.019	12	/
Stacey	2011	Netherlands	Caucasian	Prostate cancer	PB	Taqman assay	1,085	1,796	0.015	12	0.53
Stacey	2011	UK	Caucasian	Prostate cancer	HB	Taqman assay	521	1,407	0.014	11	0.60
Stacey	2011	Romania	Caucasian	Prostate cancer	PB	Taqman assay	639	815	0.008	12	0.82
Stacey	2011	USA	Caucasian	Prostate cancer	HB	Taqman assay	1,454	1,293	0.007	11	0.80
Stacey	2011	Spain	Caucasian	Prostate cancer	PB	PCR-RFLP	785	1,787	0.003	12	0.91
Stacey	2011	Iceland	Caucasian	Glioma	PB	Illumina snp chip	207	45,081	0.019	12	/
Stacey	2011	USA	Caucasian	Glioma	HB	Illumina snp chip	1,188	856	0.011	11	0.74
Stacey	2011	Iceland	Caucasian	Colorectal adenoma	PB	Illumina snp chip	4,095	43,222	0.019	12	/
Stacey	2011	Netherlands	Caucasian	Colorectal cancer	PB	Illumina snp chip	464	1,796	0.015	12	0.53
Stacey	2011	Spain	Caucasian	Colorectal cancer	PB	Illumina snp chip	184	1,940	0.003	12	0.89
Stacey	2011	Sweden	Caucasian	Colorectal cancer	PB	Illumina snp chip	1,781	1,737	0.019	12	0.42
Stacey	2011	USA	Caucasian	Colon cancer	PB	Illumina snp chip	475	807	0.004	12	0.90
Stacey	2011	USA	Caucasian	Rectal cancer	PB	Illumina snp chip	942	922	0.013	12	0.69
Stacey	2011	Iceland	Caucasian	Breast cancer	PB	Illumina snp chip	3,253	39,261	0.019	12	/
Stacey	2011	Netherlands	Caucasian	Breast cancer	PB	Illumina snp chip	725	1,794	0.015	12	0.53
Stacey	2011	Spain	Caucasian	Breast cancer	PB	Illumina snp chip	1,007	1,940	0.003	12	0.89
Stacey	2011	Iceland	Caucasian	Melanoma	PB	Illumina snp chip	724	41,073	0.019	12	/
Stacey	2011	Netherlands	Caucasian	Melanoma	PB	Illumina snp chip	683	1,796	0.015	12	0.53
Stacey	2011	Spain	Caucasian	melanoma	PB	Illumina snp chip	1,113	3,775	0.005	12	0.78

### Quantitative synthesis

**Figure 2:**
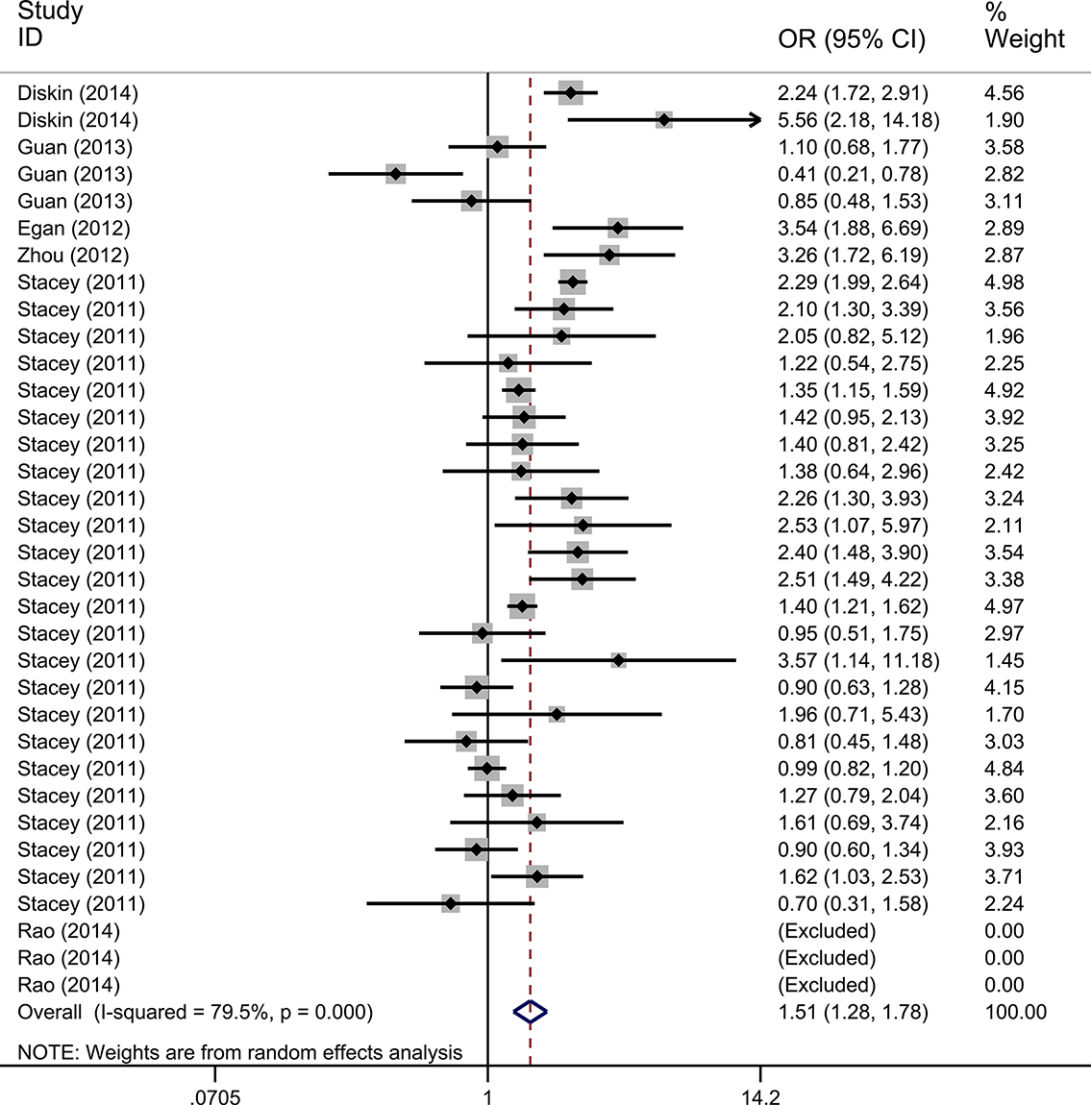
Forest plot of the association between *TP53* rs78378222 and cancer risk under heterozygous model (AC vs. AA) The estimation of OR and 95% CI of each study is plotted by a box and a horizontal line. ◊, pooled ORs and the corresponding 95% CIs.

**Figure 3:**
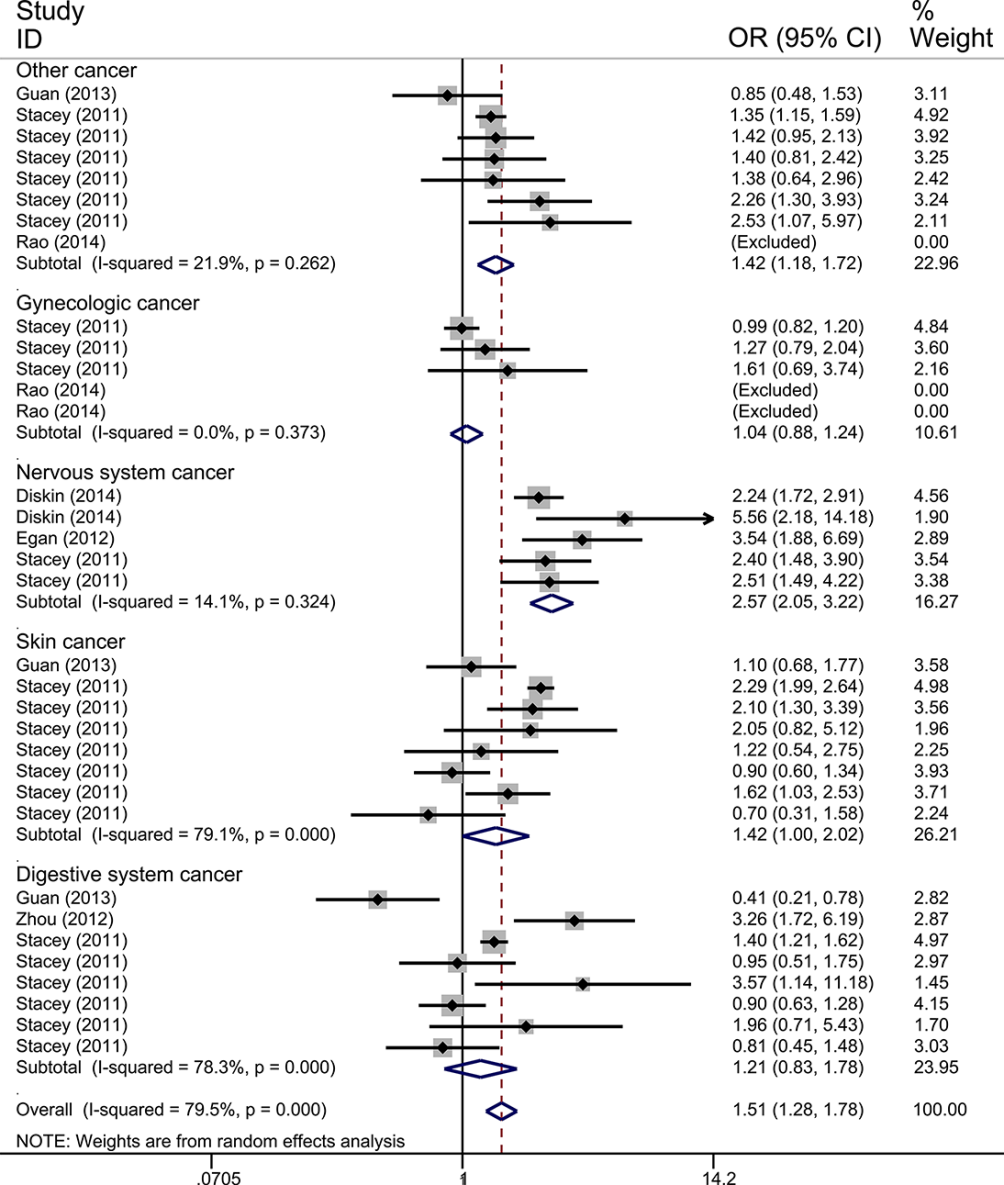
Forest plot of the association between *TP53* rs78378222 and cancer risk which is straitified by the type of cancer The estimation of OR and 95% CI of each study is plotted by a box and a horizontal line. ◊, pooled ORs and the corresponding 95% CIs.

**Figure 4:**
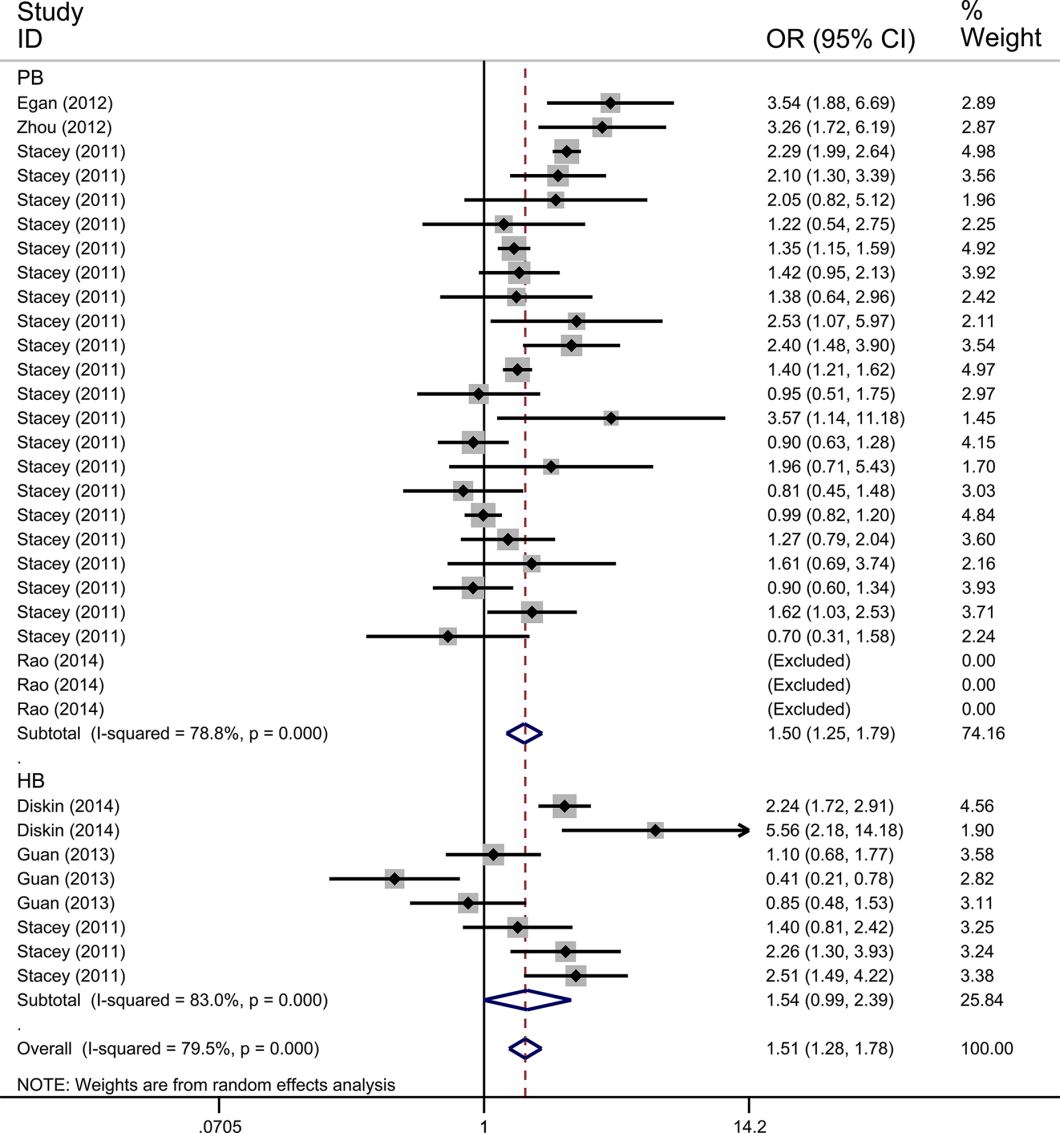
Forest plot of the association between *TP53* rs78378222 and cancer risk which is straitified by the source of controls The estimation of OR and 95% CI of each study is plotted by a box and a horizontal line. ◊, pooled ORs and the corresponding 95% CIs.

We only performed the pooled analysis under the heterozygous model (AC vs. AA). Since *TP53* rs78378222 variant homozygotes (CC) were very rare in cases and controls, we were not able to calculate risk estimates under the homozygous (CC vs. AA), dominant (AC/CC vs. AA), and recessive (CC vs. AC/AA) models. Pooled risk estimates revealed a statistically significant association between *TP53* rs78378222 and overall cancer risk (AC vs. AA: OR = 1.511, 95% CI = 1.285–1.777, *P* < 0.001) (Figure 2). The stratified analysis by cancer type revealed that *TP53* rs78378222 C allele was significantly associated with an increased risk of nervous system cancer (OR = 2.567, 95% CI = 2.046-3.222, *P* < 0.001), skin cancer (OR = 1.424, 95% CI = 1.002–2.025, *P* = 0.049), and other cancer (OR = 1.422, 95% CI = 1.176–1.721, *P* < 0.001) (Figure 3). Furthermore, in the stratified analysis by ethnicity, a statistically significant association was observed among Caucasians (OR = 1.438, 95% CI = 1.223–1.690, *P* < 0.001). Although increased cancer risk was observed among Africans and Asians, both subgroups only included one study. Thus the significance of association between rs78378222 and cancer risk among Africans and Asians was needed further validation in large well-designed studies. We also conducted the stratified analysis by source of control. Risk estimates showed a statistically significant association in the PB subgroup (OR = 1.497, 95% CI = 1.253–1.789, *P* < 0.001) but not in HB group (OR = 1.540, 95% CI = 0.992–2.393, *P* = 0.054] (Figure 4). At last, when these studies were stratified by quality score, a increased cancer risk associated with *TP53* rs78378222 polymorphism was observed in both high quality (OR = 1.406, 95% CI 1.192–1.658, *P* < 0.001) and low quality group (OR = 2.949, 95% CI = 1.839–4.728, *P* < 0.001) (Table 2).

**Table 2:** Meta-analysis of the association between rs78378222 and overall cancer risk

Variables	No. of studies	Sample size Case/control	OR (95% CI) AC vs. AA	*P*^OR^	*I*^2^(%)	*P*^heterogeneity^
All^a^	34	36,599/90,264	**1.511 (1.285–1.777)**	**< 0.001**	79.5	**< 0.001**
Cancer type						
Digestive System Cancer	7	8,346/8,012	1.211 (0.826–1.777)	0.327	78.3	**< 0.001**
Gynecologic Cancer	5	5,328/4,238	1.045 (0.882–1.239)	0.612	0.0	0.373
Nervous System Cancer	5	4,762/53,986	**2.567 (2.046–3.222)**	**< 0.001**	14.1	0.324
Skin Cancer	8	8,168/14,770	**1.424 (1.002–2.025)**	**0.049**	79.1	**< 0.001**
Other cancer	9	9,995/9,258	**1.422 (1.176–1.721)**	**< 0.001**	21.9	0.262
Ethnicity						
Caucasian	29	35,390/86,459	**1.438 (1.223–1.690)**	**< 0.001**	79.0	**< 0.001**
Africans	1	365/2,491	**5.560 (2.180–14.180)**	**< 0.001**	–	–
Asians	1	405/810	3.265 (1.723–6.187)	**< 0.001**	–	–
Indians	3	439/504	–	–	–	–
Source of control						
HB	8	9,402/14,460	1.540 (0.992–2.393)	0.054	83.0	**< 0.001**
PB	26	27,197/75,804	1.497 (1.253–1.789)	**< 0.001**	78.8	**< 0.001**
Quality score						
< 10 (low)	6	3645/8,760	**2.949 (1.839–4.728)**	**< 0.001**	51.8	0.126
≥ 10 (high)	28	32,954/81,504	**1.406 (1.192–1.658)**	**< 0.001**	78.2	**< 0.001**

HB, Hospital based; PB, Population based.

*Q* test and *I*^2^ statistic were applied to evaluate the between-study heterogeneity. There was significant heterogeneity observed in the overall analysis (*P* < 0.001, *I* ^2^ = 79.5%). Therefore, the random-effects model was selected since it generated wider CIs while calculating risk estimates. We conducted meta-regression to explore the source of heterogeneity by cancer type, ethnicity, source of control, and study quality. As shown in Table 3, the ethnicity significantly contributed to heterogeneity (*P* = 0.004), but not cancer type (*P* = 0.553) and source of controls (*P* = 0.639) in this meta-analysis.

**Table 3:** Meta-regression analysis of the main characteristics of the 34 studies

Study characteristics	Coef.	Std. Err.	*t*	*P*	95%CI	
Cancer type	0.07	0.12	0.60	0.553	−0.17	0.31
Ethnicity	1.42	0.45	3.16	**0.004**	0.50	2.33
Source of controls	0.18	0.39	0.47	0.639	−0.61	0.98

### Sensitivity analysis

**Figure 5:**
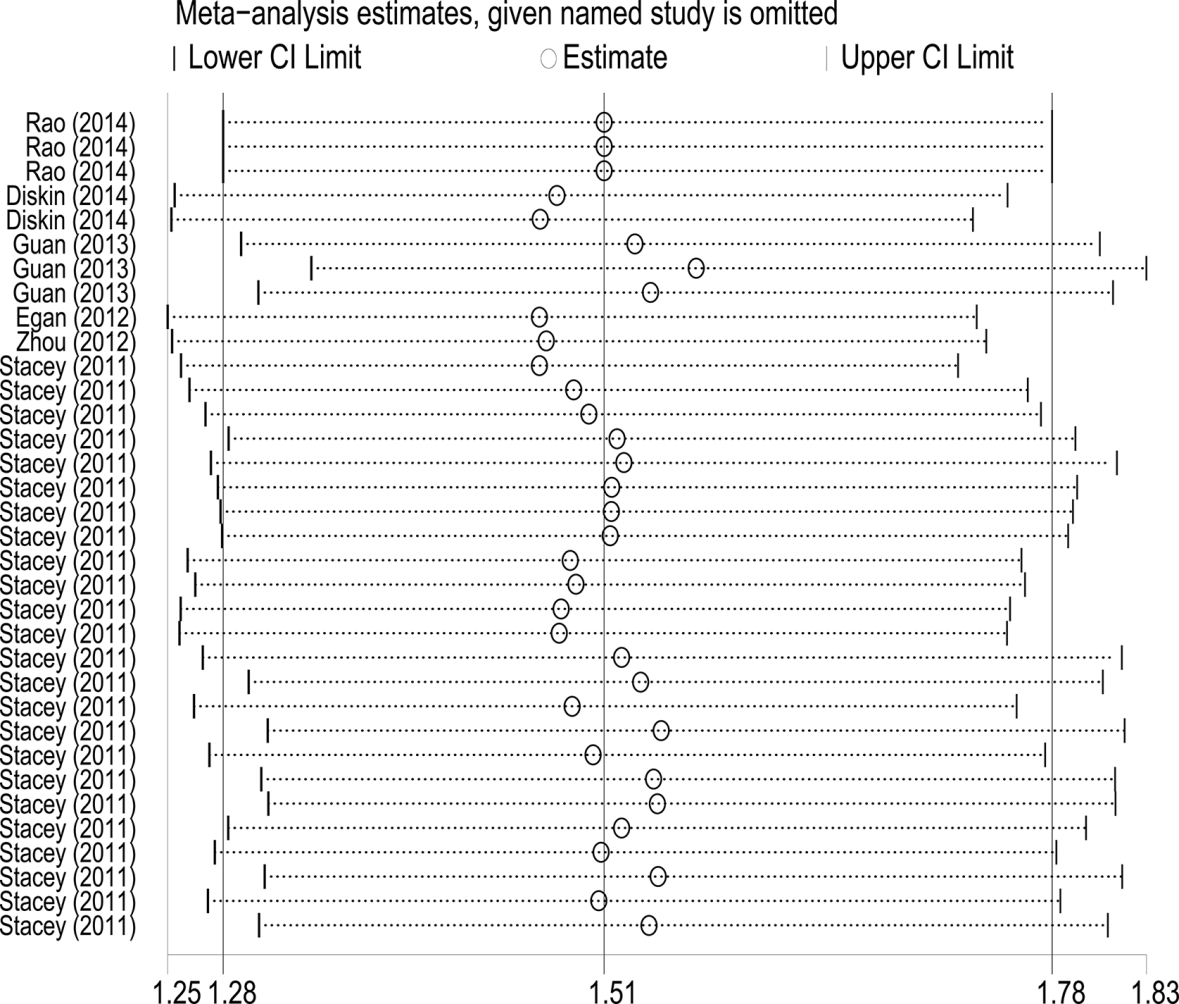
Sensitivity analysis of the association between *TP53* rs78378222 and overall cancer risk Each point represents the recalculated OR after deleting a separate study.

To evaluate the influence of individual study on the pooled ORs, we excluded one study at each time, then recalculated ORs and 95% CIs. As a result, we found that none of single study substantially changed the corresponding pooled ORs and 95% CIs (Figure 5). The sensitivity analysis indicated that our analysis was statistically robust.

### Publication bias

**Figure 6:**
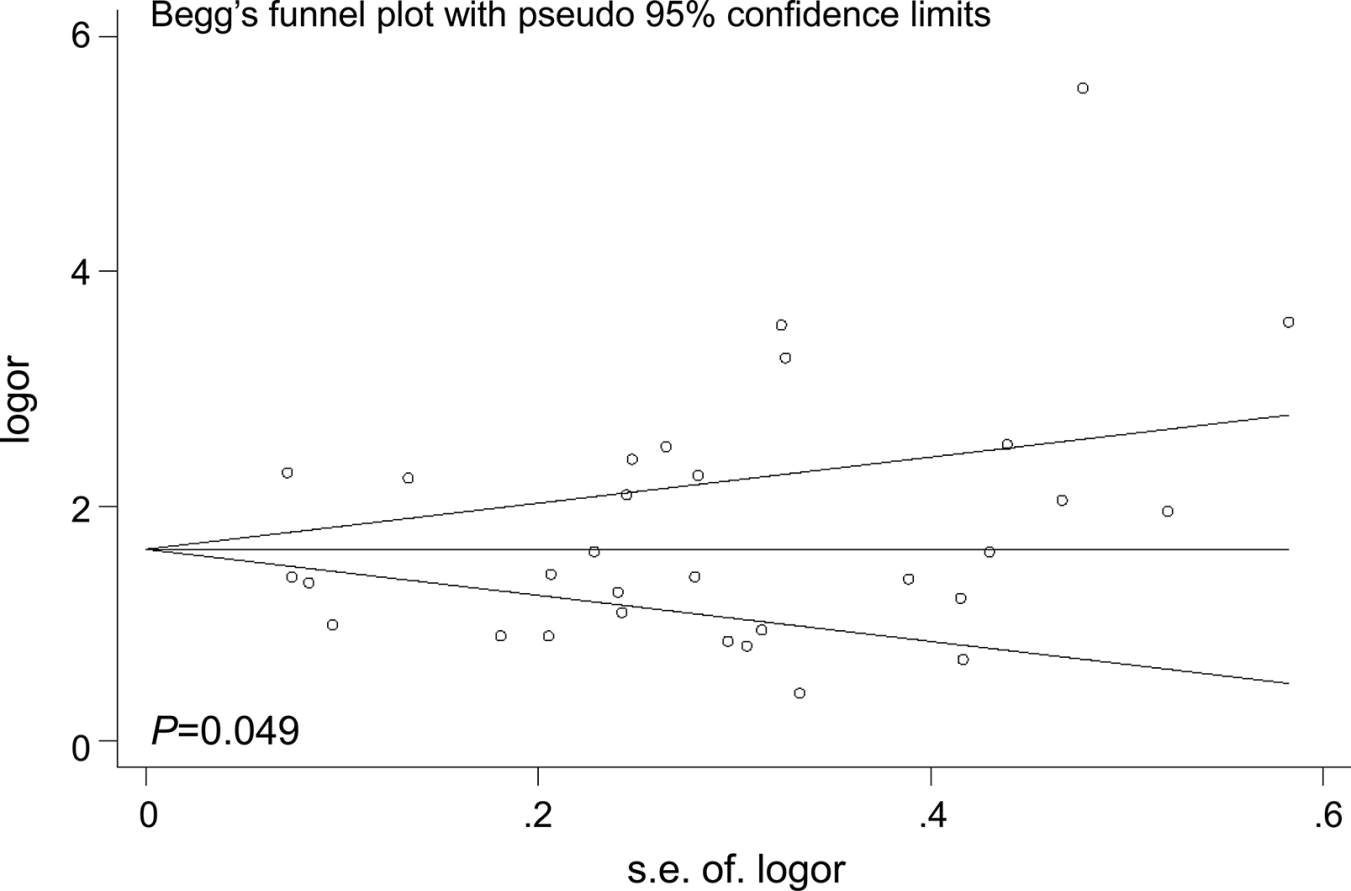
Funnel plot analysis to detect the publication bias for *TP53* rs78378222 and overall cancer risk Each point represents a separate study.

**Figure 7:**
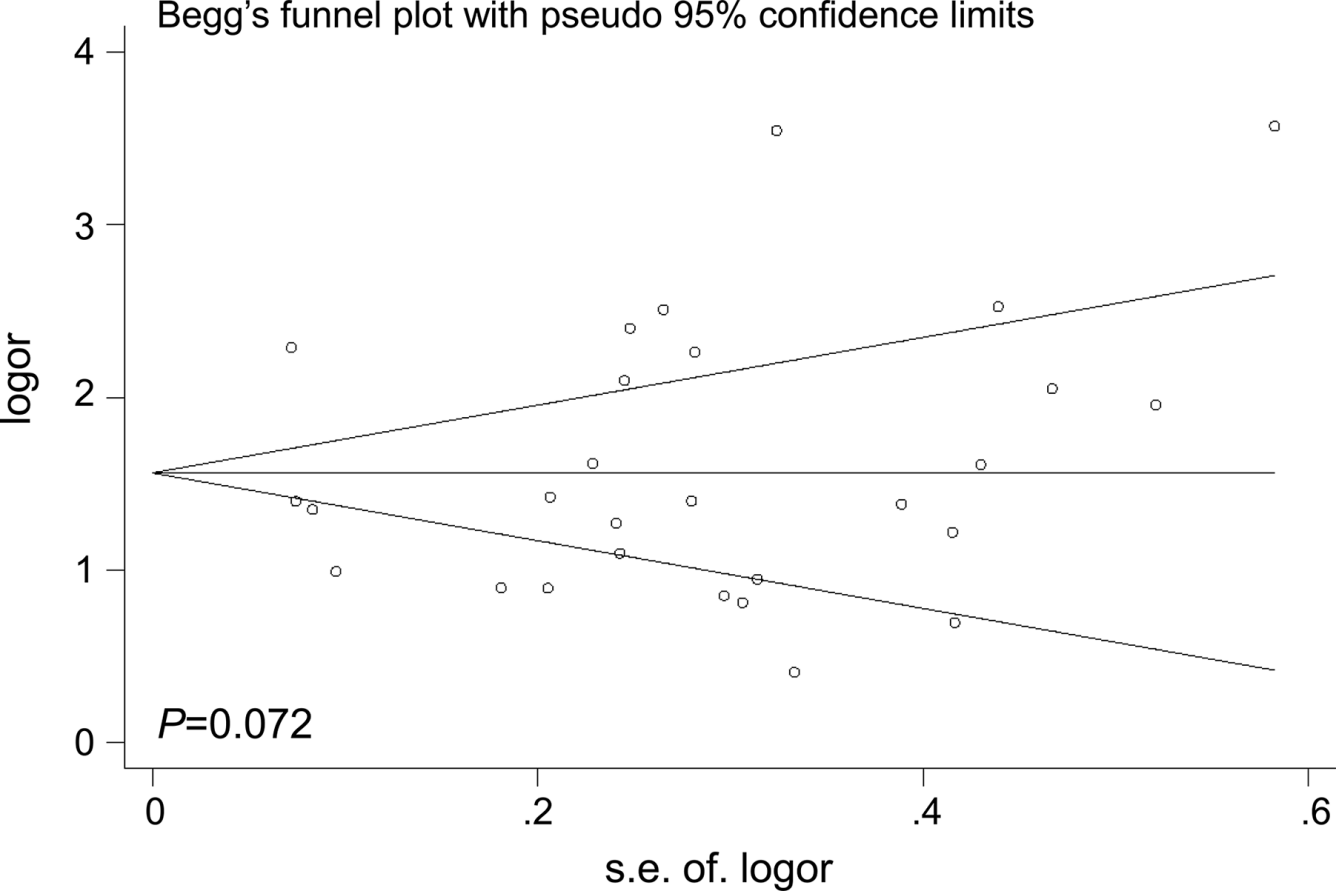
Funnel plot analysis to detect the publication bias for *TP53* rs78378222 and overall cancer risk after dropping the studies of low quality Each point represents a separate study.

The Begg's funnel plot was performed to examine the publication bias in the meta-analysis (Figures 6–7). The shape of the funnel plots appeared to relatively symmetrical. However, there was evidence of significant publication bias as indicated by Begg's and Egger's linear regression test (*P* = 0.049). Interestingly, publication bias disappeared (*P*= 0.072) when we dropped the low quality studies. These results suggested that the publication bias might be, in part, caused by those studies with poor genotyping method or selectively reported positive results.

## DISCUSSION

This meta-analysis indicated that *TP53* rs78378222 polymorphism was significantly associated with overall cancer susceptibility. Furthermore, stratification analysis by ethnicity suggested that the AC genotype of rs78378222 conferred cancer susceptibility among Caucasians, Africans, and Asians. Moreover, while stratified analysis were carried out by cancer type, source of controls, and quality score, significant association were identified in nervous system cancer and other cancer subgroup, HB subgroup, high score and low score subgroup. To the best of our knowledge, this is the first meta-analysis to evaluate the association between *TP53* rare variant-rs78378222 and overall cancer susceptibility, and the sample size of the meta-analysis was relatively large. with a total of 127,871 subjects.

The *TP53* gene is composed of 11 exons and 10 introns, encoding tumor suppressor protein p53. While DNA damage occurs, p53 is involved in determining to repair the damaged DNA or initiate apoptosis. It inhibits cell proliferation via keeping cells from excessively dividing and growing. Numerous evidence substantiated that inherited variants in the *TP53* gene notably increased the risk of developing cancer, such as Li-Fraumeni mutations [6]. It was reported that a woman with a novel 7–9 exons deletion on *TP53* presented early-onset breast and ovarian cancer and subsequently developed acute myeloid leukemia [21]. Recently, rare variants were found presenting a more important functional effect than do common variants [12, 13]. The rare germline variant rs78378222 is a newly found SNP in a GWAS by Stacey in 2011 [15]. A number of evidences suggested that this variant increased the risk of prostate cancer, glioma and other cancers and might correlate to a unfavorable prognosis [3, 15, 16, 17, 22]. However, there was no formal meta-analysis about this important variant before. Then we conducted this meta-analysis to systematically evaluate the association between rs78378222 polymorphism and cancer susceptibility. The quality of data set from GWAS or high-throughput sequencing is generally higher than that of single polymorphism detection. Moreover, the larger sample sizes and the large-scale validation in GWASs ensures the reliability of the results. Including these high-grade and more credible evidence would make our meta-analysis sense.

The results revealed a significant association between *TP53* rs78378222 polymorphism and overall cancer risk. Specifically, stratified analysis revealed that this rare variant increased susceptibility to nervous system cancer, skin cancer and other cancer. The ORs (95% CIs) between different subgroup varied greatly from 1.045 (0.882–1.239) to 2.567 (2.046–3.222). SNPs in a gene are typically cancer-specific. The discrepancy in ORs between different cancers might reflect the inherent heterogeneity of oncogenic progression in different cancer types [23, 24]. In addition, a significant increased risk was observed among PB subgroup, but not among HB subgroup. Lack of association among HB subgroup was probably due to the fact that the controls recruited from hospital couldn't represent the general population well. At last, although subgroup analysis by ethnicity revealed the association among Asians and Africans, these data should be interpreted with caution. Since there was only one study included in either subgroup, the result may be false positive and unstable.

Stratification analyses and meta-regression indicated the between-study heterogeneity in the overall analysis was due to ethnicity. The ethnicity-dependent association might be attributed to the distinction in genotype frequency between controls of different ethnic groups. Cancer is a complicated disease and results from gene-environment interaction. Therefore, different genetic backgrounds among different races could help to interpret the ethnicity-dependent data. For example, different continental populations usually have different linkage disequilibrium patterns. The *TP53* rs78378222 polymorphism may be in close linkage disequilibrium with a causal variant in one population but not in another. Unexpectedly, there was heterogeneity observed among the same population-Caucasians in this meta-analysis. This might be caused by genetic heterogeneity between racial characters of species, although these persons all belonged to Caucasians. For instance, subjects from Iceland in Stacey's study [15] are different from other Caucasians. On the other hand, clinical features or lifestyle may also help to explain the heterogeneity of ethnicity.

The current analysis might have the following advantages: (i) this study is the first systematical meta-analysis regarding association between rs78378222 and overall cancer risk; (ii) rs78378222 is a newly reported rare variant on *TP53* in recent years and the included data was mostly from GWAS and high-throughput sequencing, which was more credible; (iii) the sample size is very large (127,871) and the subjects are mostly selected from multi-center cancer registry community. Thus, this analysis might provide more potent statistical power; (iv) this meta-analysis included the latest literatures till Nov, 2015 to ensure the comprehensiveness and minimize the selection bias. Although this is the first comprehensive meta-analysis about relationship between rs78378222 and overall cancer risk, several limitations should be addressed. First, the stratified analyses in some subgroup analysis, like among Africans and Asians (< 5 studies), might have insufficient statistical power to assess the real association. Second, our analysis was on the basis of ORs estimated without adjustment for several potential confounding factors, because there was little information about smoking, drinking status, and carcinogen and radiation exposure, which are known to have major effect on the carcinogenesis. The absence of valuable data might result in confounding bias and limit the evaluation of gene-environment interactions. The third, selection bias could exist because researchers were prone to report positive data, and the articles retrieved from NCBI or EMBASE were published in English only. Overall, due to these limitations, the finding of this investigation should be interpreted with caution.

In summary, this systematical meta-analysis indicated that *TP53* rare variant-rs78378222 significantly increased the risk of cancer. In addition, the significant association between *TP53* rs78378222 and cancer risk was observed in PB studies and both high score and low score studies. The well-designed, multi-center and large-cohort studies are needed to confirm our findings.

## MATERIALS AND METHODS

The literature search, data collection and articles inclusion of this meta-analysis were performed following the latest meta-analysis guidelines (PRISMA) [25].

### Identification of the eligible studies

A comprehensive literature search of PubMed and EMBASE was undertaken without language restriction. The retrieval items included “*TP53* rare or rs78378222”, “polymorphism or mutant or variant”, and “cancer or tumor or carcinoma”. The retrieved studies included original researches, review articles and other relevant studies. If studies were performed with overlapping data, only the latest or the largest studies would be included in this meta-analysis. We further searched China National Knowledge infrastructure (CNKI) and Chinese Biomedical (CBM) database for more eligible studies in Chinese. Finally, we manually searched the references of bibliographies and potential relevant literatures to find more eligible studies.

### Inclusion criteria

All the studies included in the current analysis should meet the following criteria: (i) case-control design; (ii) providing enough information to estimate ORs and the corresponding 95% CIs; (iii) investigating the association between rare *TP53* rs78378222 variant and cancer risk; (iv) Observed genotype frequencies in controls were in agreement with Hardy-Weinberg equilibrium(HWE), or there is evidence that another polymorphism in the *TP53* gene was in compliance with HWE.

The exclusion criteria were: (a) case reports; (b) case only studies; (c) conference abstracts; (d) review articles; (e) non-cancer subjects only studies; (f) duplicate publications.

### Data extraction

Two authors (Y.W. and J.H.) independently reviewed the articles carefully and extracted the detailed information from each study. All the conflicts were resolved by full discussion until a consensus was reached. The following information was collected: year of publication, first author's surname, ethnicity, country of origin, cancer type, the source of controls, *P*-value of HWE in controls, genotyping methods, the matching level between cases and controls, genotype counts of cases and controls for rs78378222, total number of cases and controls.

The stratified analysis was performed by ethnicity (Caucasians, Africans, Asians and Indians), cancer type (digestive system cancer, nervous system cancer, skin cancer, gynecologic cancer, other cancer), the source of control (HB: hospital-based controls; PB: population-based controls) and quality score (low quality: < 10; high quality: ≥ 10). Publications were classified to different studies if they contained subjects with different cancer types, ethnics and so on.

### Quality score assessment

The quality of eligible was independently assessed by two investigators (Y.W. and J.H.) based on the quality assessment criteria (Table S1) [26, 27]. The evaluation items were as follows: representativeness of case, representativeness of control, ascertainment of cancer, control selection, genotyping examination, HWE, and total sample size. Each study was evaluated on a scale from 0–15. All studies were classified as “low quality” (score < 10) or “high quality” studies (score ≥ 10).

### Statistical method

The strength of association between rs78378222 and cancer risk was evaluated by calculating the crude ORs and 95% CIs. For *TP53* rs78378222 polymorphism, the pooled OR was performed under the heterozygous model. *Z* test were applied to confirm the statistical significance of an association. The Cochran *Q*-test and *I*^2^ statistics were used to assess between-study heterogeneity. For *Q*-test, a *P* value < 0.10 indicated there was statistically significant heterogeneity in the meta-analysis, and a random-effect model was used. Otherwise, a fixed-effect model was performed. *I*^2^ represented the proportion of variation in the meta-analysis attributed to heterogeneity among studies. The leave-one-out sensitivity analysis was conducted by sequentially excluding a study at each time and recalculating ORs. Moreover, the publication bias was assessed by Begg's and Egger's linear regression test and funnel plot [20]. Finally, a meta-regression was conducted to detect the main sources of heterogeneity in the meta-analysis.

All statistical analysis was performed using the STATA version 12.0 (STATA Corporation, College Station, TX). All the *P* values were two-sided. A *P* value of < 0.05 was considered statistically significant.

## SUPPLEMENTARY MATERIALS TABLE



## Abbreviations

TP53: tumor protein 53
GWAS: Genome-wide association study
OR: odds ratio
95% CI: 95% confidence interval
SNP: single nucleotide polymorphism
CBM: Chinese Biomedical
CNKI: China National Knowledge infrastructure
HWE: Hardy-Weinberg equilibrium
HB: hospital-based controls
PB: publication-based controls
